# Zinc Deficiency Leads to Lipofuscin Accumulation in the Retinal Pigment Epithelium of Pigmented Rats

**DOI:** 10.1371/journal.pone.0029245

**Published:** 2011-12-22

**Authors:** Sylvie Julien, Antje Biesemeier, Despina Kokkinou, Oliver Eibl, Ulrich Schraermeyer

**Affiliations:** 1 Section of Experimental Vitreoretinal Surgery, Centre of Ophthalmology, Institute for Ophthalmic Research, Tuebingen, Germany; 2 Institute of Applied Physics, Eberhard Karls University of Tuebingen, Tuebingen, Germany; University of Florida, United States of America

## Abstract

**Background:**

Age-related macular degeneration (AMD) is associated with lipofuscin accumulation whereas the content of melanosomes decreases. Melanosomes are the main storage of zinc in the pigmented tissues. Since the elderly population, as the most affected group for AMD, is prone to zinc deficit, we investigated the chemical and ultrastructural effects of zinc deficiency in pigmented rat eyes after a six-month zinc penury diet.

**Methodology/Principal Findings:**

Adult Long Evans (LE) rats were investigated. The control animals were fed with a normal alimentation whereas the zinc-deficiency rats (ZD-LE) were fed with a zinc deficient diet for six months. Quantitative Energy Dispersive X-ray (EDX) microanalysis yielded the zinc mole fractions of melanosomes in the retinal pigment epithelium (RPE). The lateral resolution of the analysis was 100 nm. The zinc mole fractions of melanosomes were significantly smaller in the RPE of ZD-LE rats as compared to the LE control rats. Light, fluorescence and electron microscopy, as well as immunohistochemistry were performed. The numbers of lipofuscin granules in the RPE and of infiltrated cells (Ø>3 µm) found in the choroid were quantified. The number of lipofuscin granules significantly increased in ZD-LE as compared to control rats. Infiltrated cells bigger than 3 µm were only detected in the choroid of ZD-LE animals. Moreover, the thickness of the Bruch's membrane of ZD-LE rats varied between 0.4–3 µm and thin, rangy ED1 positive macrophages were found attached at these sites of Bruch's membrane or even inside it.

**Conclusions/Significance:**

In pigmented rats, zinc deficiency yielded an accumulation of lipofuscin in the RPE and of large pigmented macrophages in the choroids as well as the appearance of thin, rangy macrophages at Bruch's membrane. Moreover, we showed that a zinc diet reduced the zinc mole fraction of melanosomes in the RPE and modulated the thickness of the Bruch's membrane.

## Introduction

Age-related macular degeneration (AMD), a disease that typically affects both eyes at different rates, is the leading cause of irreversible blindness among Caucasians over the age of 65 in the Western world [1]–[3]. The specific pathogenic causes of macular degeneration are multi-complex and poorly understood. A large number of risk factors like smoking, obesity, race, family history, gender, nutrition, several diseases and systemic vascular disorders are still under investigation but the greatest proved risk factor for AMD is aging.

AMD is more prevalent in white than in black populations [1], [3]–[4]. In addition, primary lesions associated with loss of vision in AMD are believed to be located in the retinal pigment epithelium (RPE) [5]. The content of melanosomes in RPE cells decreases and melanosomes undergo age-related changes while the amount of lipofuscin and melanolipofuscin granules increases [6]–[8].

Melanin as part of the melanosomes is believed to play a protective role for the retina based on its ability to screen light from sensitive tissues [9], or by sequestering heavy metals that catalyze oxidative reactions [10], and by trapping free radicals produced by photochemical radiation [11]. Paradoxically, melanin is also known to produce free radicals and to oxidize physiological substrates during ultraviolet and visible light exposure [12]–[15]. Furthermore, melanin precursors and melanin itself can be considered as a free radical [16], [17].

Zinc is an essential trace element that occurs in high concentrations in pigmented tissues like the choroid and there especially inside the melanosomes [18]. It is known to participate as a cofactor of several antioxidant enzymes [19], to be involved in the visual cycle in dependence with the retinol dehydrogenase and rhodopsin regeneration [20] and to play a crucial role in the metabolism of ingested photoreceptor outer segments in the RPE cells [21]. For many years, a link between low zinc levels and AMD was proposed [22]–[25]. Consistent with this hypothesis, macular zinc levels were found to be decreased in AMD patients [26]. Furthermore, in some but not all studies, oral zinc supplementation slowed the progression of AMD [23], [27]. However, it is yet unclear how the deficiency of zinc may contribute to the pathogenesis of AMD. Since one of the pathological features of AMD is retinal cell degeneration and since zinc depletion causes cell death in various cell systems [28], in the present study, we investigated the morphological and ultrastructural effects of zinc deficiency in pigmented rat eyes by keeping animals six months in a zinc-free status.

## Results

### 1) Assessment of zinc deficiency

**Figure 1:**
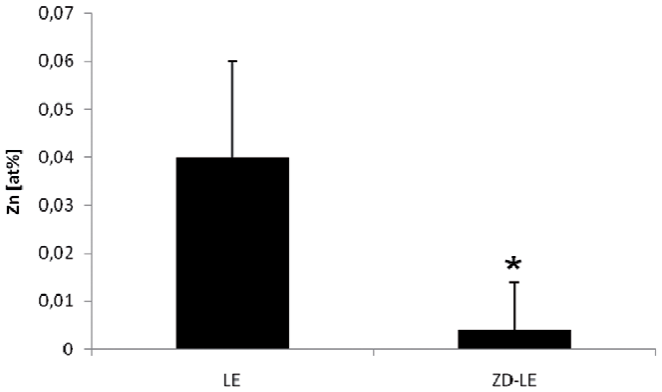
Zinc mole fraction (in at%) of melanosomes in the RPE of control LE and ZD-LE animals as determined by quantitative EDX spectroscopy in the TEM. In the ZD-LE group (0.004±0.01 at% zinc) which is below the detection limit of 0.02 at%, the zinc mole fractions were significantly lower compared to the control group (0.04±0.02 at% zinc) (p = 0.02, t test).

The chemical composition of RPE melanosomes was analysed using EDX. In LE rats, the melanosomes of the RPE contained 0.03–0.07 at% Zn (mean value 0.04±0.02 at%). In ZD-LE rats, the zinc mole fractions were always at or below the minimum detectable mole fraction of 0.02 at% (0.004±0.01 at%) and therefore significantly lower (p = 0.02) compared to controls (Fig. 1).

### 2) Fluorescence microscopy

**Figure 2:**
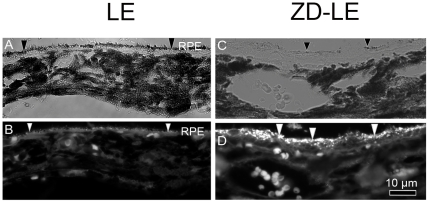
Light microscope and fluorescence micrographs of LE and ZD-LE rats. **A**) Bright-field image of melanin pigmentation in LE rat. **B**) Auto-fluorescence of lipofuscin and melanolipofuscin pigmentation of the same area as in (A). **C**) Bright-field image of melanin pigmentation in ZD-LE rat. **D**) Auto-fluorescence of lipofuscin and melanolipofuscin pigmentation of the same area as in (C). In LE rats, the RPE contains a high content of melanosomes (black arrowheads, A) and a low content of lipofuscin (white arrowheads, B). In contrast, in ZD-LE rats, the RPE contains a low content of melanosomes (black arrowheads, C) and a high content of lipofuscin (white arrowheads, D).

Figure 2 shows the RPE/choroid interface of control LE rats (A, B) and ZD-LE rats (C, D) as bright-field (A, C) and fluorescence (B, D) images. Under the fluorescent microscope, the lipofuscin and melanolipofuscin granules were identified by their auto-fluorescence (Figs. 2B, D) but could not be distinguished. Therefore, the quantification of lipofuscin granules was performed by electron microscopy; however, the fluorescent micrographs show clearly an increase of auto-fluorescent granules in the RPE from the ZD-LE animals compared to the LE control rats (Figs. 2B and 2D). Moreover, the bright-field images show a high content of melanosomes in the RPE of LE rats (Fig. 2A) whereas it was lower in the RPE of ZD-LE animals (Fig. 2C).

### 3) Quantification of lipofuscin granules by electron microscopy

**Figure 3:**
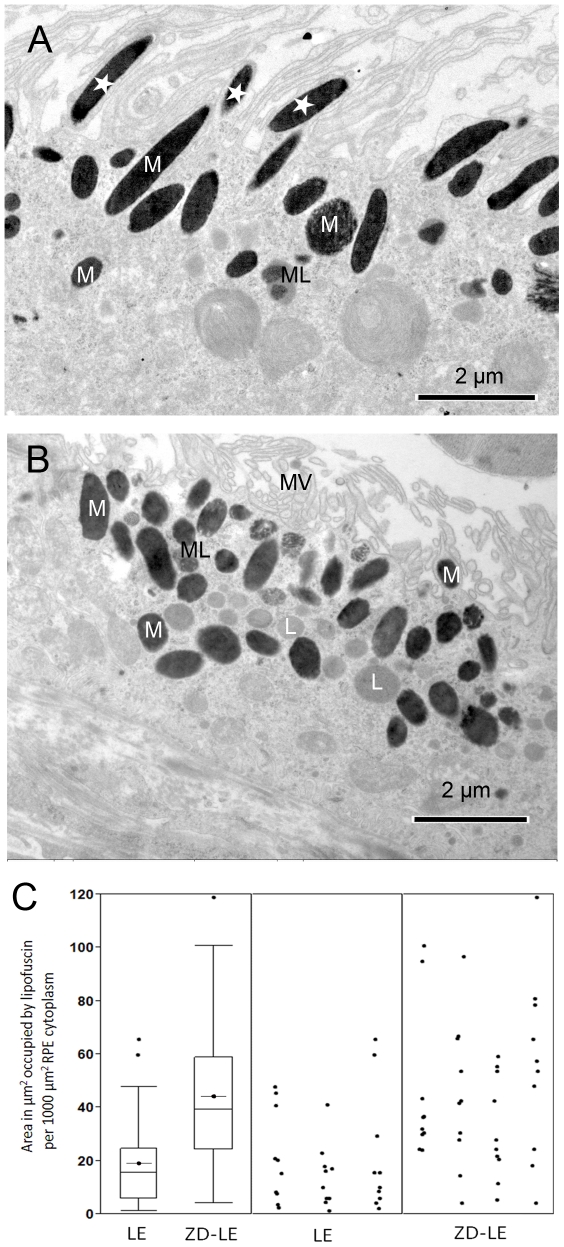
TEM micrographs of RPE cells of A) LE rat and of B) ZD-LE rat showing melanosomes (M), melanolipofuscin (ML) and lipofuscin (L) granules. In (B), note the rarefaction of melanosomes found normally in the RPE microvilli as seen in (A) (white asterisks). Note the crooked microvilli (MV) which covered a smaller area than typical upright positioned microvilli in control animals in (A). **C**) Area fraction of lipofuscin granules in the RPE layer of LE and ZD-LE animals at ultrastructural level by using the iTEM image analysis software. The left side of the figure shows box plots with whiskers from minimum to maximum, the medians, outliers and also the means of the data (LE: 19±18 µm^2^ vs ZD-LE: 44±28 µm^2^, p<0.0001, Mann-Whitney test). The right side of the figure shows separately the quantification of the lipofuscin granules in each animal (three LE rat eyes and four ZD-LE rat eyes). Ten micrographs per eye were used to determine the area fraction of lipofuscin expressed as area in µm^2^ occupied by lipofuscin per 1000 µm^2^ RPE cytoplasm.

Under the electron microscope, the lipofuscin granules can be identified as spheroidal or lamellar organelles which exhibit a homogenous gray matrix (Fig. 3A–B). The total areas (in µm^2^) occupied by lipofuscin per 1000 µm^2^ sectioned RPE cytoplasm clearly increased in ZD-LE animals (44±28 µm^2^) compared to the LE control rats (19±18 µm^2^) (Fig. 3C). Application of the Mann-Whitney test revealed that this increase is statistically significant (p<0.0001).

### 4) Ultrastructural observations in the TEM

**Figure 4:**
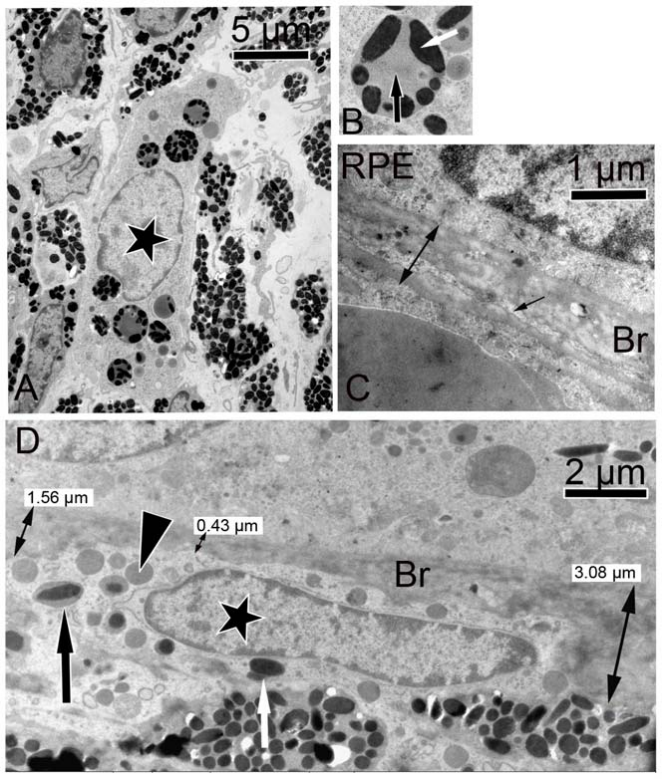
TEM micrographs of ZD-LE eyes. A) a macrophage in the choroid (black asterisk, also seen in figs. 5B and 6D) contains melanosomes. **B**) a higher magnification of the cell seen in (A) bearing lysosomes containing homogenous material (black arrow) and melanosomes (white arrow). **C**) Bruch's membrane (Br) delimitated on one side by the basal labyrinth of the RPE and on the other side by a capillary (double arrow). The arrow shows an infiltrated cell inside the Bruch's membrane. The five layers of the Bruch's membrane are no more identifiable. **D**) Choroid-RPE interface showing a thin macrophage just below the Bruch's membrane (black asterisk in the nucleus, also seen in fig. 6D) which contains lipofuscin- (black arrowhead), melanin- (white arrow) and melanolipofuscin-like (black arrow) granules. Note the varying thickness (0.4–3 µm) of the Bruch's membrane (Br) and the increase of the extracellular matrix in Bruch's membrane on both sides of the image.

The retina, RPE and choroid tissues were examined to determine ultrastructural differences in ZD-LE rats compared to the LE controls. The melanosomes were homogenous distributed over the whole area of the RPE in each of the animals. The amount of oval melanosomes was not different. However, the amount of melanosomes in the microvilli was significantly decreased (p<0.001) in ZD-LE rats (19±9) compared to LE rats (90±3) independent of shape. This effect might be due to the fact that ZD-LE animals show crooked microvilli (Fig. 3A–B) which covered a smaller area (272±85 µm) than typical upright positioned microvilli in control animals (529±15 µm) (p = 0.004). Giant melanosomes (not shown, manuscript in preparation) and lysosomes containing homogenous material and melanosomes were detected in large round macrophages (see immunohistochemistry) of the choroid of ZD–LE rats (Fig. 4A–B). The five layers of the Bruch's membrane were not distinguishable anymore (Fig. 4C). Very thin and rangy macrophages attached at the Bruch's membrane containing melanin- and lipofuscin-like granules were observed as well (Fig. 4D). The pigment granules inside these macrophages resembled melanolipofuscin granules that are usually only present inside RPE cells (Fig. 4D). The Bruch's membrane presented a pathological aspect indicated by its irregular thickness varying from 0.4 µm to 3 µm, proliferation of the extracellular matrix and by the presence of infiltrated cells (Figs. 4C and D).

### 5) Immunohistochemistry and light microscopy

**Figure 5:**
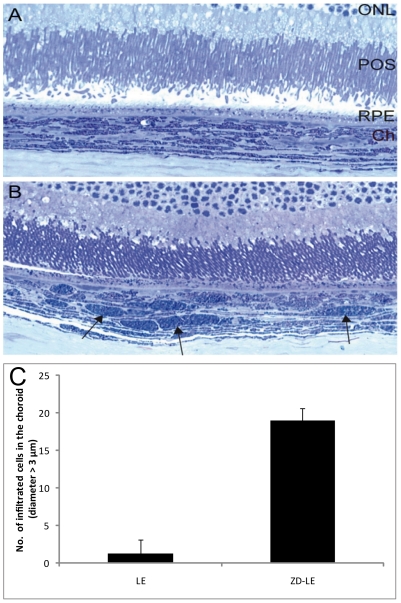
Light microscope semi-thin sections showing the retina-choroid complex of A) LE rat and of B) ZD-LE rat. In B, the choroid shows many large round cells (black arrows) which are absent in the control rats (A). **C**) Quantification of infiltrated pigmented cells with a diameter larger than 3 µm in the choroid of LE (1.3±1.9) *vs*. ZD-LE (19±1.6) rats. The increase observed in ZD-LE rats is statistically significant (p<0.0001). Abbreviations: ONL, Outer Nuclear Layer; POS, Photoreceptor Outer Segments; RPE, Retinal Pigment Epithelium; Ch, Choroid.

**Figure 6:**
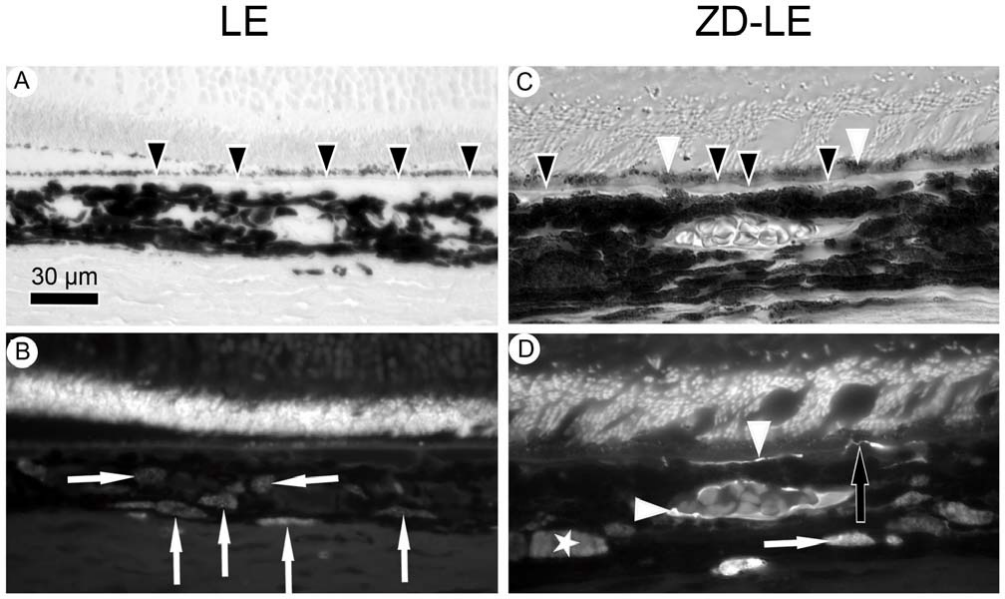
Light microscope and fluorescence micrographs of LE (A–B) and ZD-LE (C–D) rats. **A**) Bright-field image of the retina - RPE – choroid complex in LE rat. Black arrowheads show the regularity of the Bruch's membrane. **B**) Fluorescence micrograph of ED1 positive cells in the same area as in (A). ED1 immunoreactivity (white arrows) is visible in the choroid. **C**) Bright-field image of the RPE – choroid complex in ZD-LE rat. Black arrowheads show the irregularity of the Bruch's membrane, white arrowheads indicate RPE cells. **D**) Fluorescence micrograph of ED1 positive cells in the same area as in (C). Small (<3 µm) (white arrow) and large (>3 µm) (white asterisk) ED1 positive cells are detected in the choroid. ED1 immunoreactivity is also shown around choroidal blood vessels and along the choriocapillaris just below the Bruch's membrane (white arrowheads). A thin ED1 positive cell simultaneously in contact with the Bruch's membrane and a RPE cell (black arrow) is shown. In B and D), note the strong autofluorescence of the photoreceptor outer segments frequently observed in paraffin sections due to the fluorescence emitted by the retinoids reflecting the clearance of toxic byproducts and the accumulation of lipofuscin-like fluorophores associated with the increase of lipid peroxidation with age in the retina.

Four different types of macrophage-like cells were observed: 1) thin and rangy cells below (Fig. 4D) and inside (Fig. 4C) the Bruch's membrane and 2) large heavily pigmented cells (>3 µm) regionally but often found and solely in the choroid of ZD-LE animals (Figs. 4A and 5). The amount of pigmented cells, bigger than 3 µm, was counted from the optic nerve till the ciliary body (Fig. 5C). The result in ZD-LE rats (19±1.63) compared to LE controls (1.25±1.89) was found statistically significant (p<0.0001, t test). Immunohistochemically, both types were identified as ED1 positive cells (Fig. 6).

3) Small ED1 positive cells (<3 µm) were found both in the choroid of LE and ZD-LE rats (Figs. 6 B and D).

4) Moreover, ED1 immunoreactivity was detected around choroidal blood vessels and along the choriocapillaris, but only in ZD-LE rats (Fig. 6D).

## Discussion

Zinc deficiency experiments were widely carried out in the past using different diet-periods or animal models. Previous work described a great sensitivity of the rat retina [20], changes in elemental content of RPE and choroidal melanosomes by means of EDX [29], and decreased activity of alkaline phosphatase, the most sensitive indicator of zinc status [30]. The present study compliments the EDX results obtained in pigs maintained on low zinc diets [29] since it addresses also the minimum detectable mole fraction (MDMF) of EDX analysis and proves that after a low zinc diet, the Zn mole fraction decreases below the MDMF in RPE melanosomes. Both investigations observed giant roundish melanosomes in the choroid. The large melanosomes that appeared can be the result of reduced normal cellular function and/or fusion of altered melanosomes. It has been reported that a zinc deficient diet results in an “ageing-like” process, with melanosomal morphological changes [31]–[32], severe degeneration of the photoreceptor outer segments and osmiophilic inclusion bodies in the RPE [33]–[34]. Such alterations are typically found in pigmentary disorders [35]–[36].

In addition, both investigations showed cells containing melanosomes inside lysosomes. However, while Samuelson et al [32] described them as melanocytes by ultrastructure, we found that they are ED-1 positive macrophages.

Although the current study was made from a small population of animals, the trends seen in the zinc diet group were remarkably consistent, indicating that zinc deficiency nutrition yields lipofuscin accumulation in pigmented animals with concomitant involvement of ED1 positive cells, i.e. macrophages. The small ED1 positive cells (<3 µm) observed in the choroids of LE and ZD-LE rats are probably resident choroidal macrophages. Zinc deficiency is known to compromise the function of macrophages [37]. Our study indicates that lipofuscin formation is associated with the appearance of large ED1 positive cells in the choroid. In the present study, ED1 positive cells were also found around blood vessels in the choroid and in the choriocapillaris as well as directly below the Bruch's membrane and those ED1 positive cells were also able to form contact with RPE cells. Possibly, a transfer of melanosomes into the ED1 positive cells took place after chemical modification of the melanosomes after zinc deficiency. Since macrophages were closely attached to a thin (0.43 µm) Bruch's membrane and in addition contained melanolipofuscin and lipofuscin-like granules, it is more likely that these granules originated from the RPE rather than from choroidal melanocytes. In a recent study investigating the aging Ccl2-knockout mice as model of AMD, a pronounced accumulation of swollen macrophages containing pigment granules and lipofuscin inclusions was also observed, however in the subretinal space and not sub-RPE [38]. Also, we recently observed that lipofuscin was transferred from RPE to macrophages in monkey eyes after drug treatment (Schraermeyer U, et al. *IOVS* 2010;51: ARVO E-Abstract 2786). These macrophages migrated through the Bruch's membrane and took up lipofuscin granules. Moreover, in a previous work from our group, we have shown that a cellular transport of subretinal material into choroidal and scleral blood vessels involving macrophages can take place [39].

In the present study, the zinc deficiency did affect the remodelling of the Bruch's membrane, possibly by disturbing its matrix metalloproteinase system that consists of proteolytic zinc-containing enzymes [40], [41]. The penetration of macrophages from the choroid to the RPE through the Bruch's membrane might be therefore facilitated. However, a proof of this hypothesis requires further investigations.

Lipofuscinogenesis results from oxidative reactions yielding an increase of free radicals [42]. Zinc deficiency promotes lipid peroxidation thereby damaging the lipid membranes [43] and yielding phagocytic and lysosomal functional deficiency [44]–[45]. The increased formation of lipofuscin in the zinc deficient rats might be due to elevated oxidative stress and incomplete digestion of photoreceptor outer segments in the lysosomes of the RPE. Such an accumulation of lipofuscin-like products, presenting the lysosomal storage bodies in the RPE layer, can contribute to AMD [46]. It was shown that a nutrition poor in antioxidants, results in an accumulation of lipofuscin-like autofluorescence in albino rats [47]. Here, we evidenced that zinc deficiency alone can lead to accumulation of lipofuscin-like products.

The retina consists of high concentrations of polyunsaturated fatty-acids and is exposed to light and high oxygen pressure. A deficiency in micronutritions can mimic DNA damage, similar to radiation [48]. Dietary zinc deficiency results in an increase of free iron and hydrogen peroxide, increasing a free radical mediated attack on DNA, proteins and membrane lipids [49]. Iron is found to induce the accumulation of lipofuscin like material in albino Fischer rats [50]. The present experiments demonstrated that not or not only the amount of melanosomes but the adequate amount of zinc plays an important role in preventing accumulation of lipofuscin in the ocular tissues.

The RPE is a barrier for circulation products and melanosomes are the storage space for trace elements and do interact with drugs. This study shows that the metal ion concentration of melanosomes in the RPE can be regulated by the zinc content of the nutrition. The ability of the “aging” melanosomes to bind metals, including zinc, is assumed to decrease. Changes in the melanosomal properties, with reduced metal-binding ability, are involved in degenerative processes, like AMD. The zinc level of the RPE - choroid complex was found to be reduced to 24% in donors with AMD as compared to donors not being affected by AMD, suggesting that the metal homeostasis plays a role in AMD and in retinal health [51].

In our previous studies, we showed that zinc uptake in human donor eyes is influenced by the iris and fundus pigmentation [52]–[53]. We assume that melanosomes without adequate uptake of zinc change their structural and probably functional properties, which was shown before in pig eyes [31]–[32]. Here we found that this mechanism also applies for rat eyes. Moreover, lack of zinc yields lipofuscin accumulation in pigmented rats by unknown mechanisms.

## Materials and Methods

### 1) Ethics Statement

The zinc diet experiments were performed in 2003 and started at the University of Cologne (Germany). The local authorized agent for animal protection was informed and agreed that an approval for an animal experiment was not necessary because the animals were fed with a commercial available zinc deficient diet that was not harmful to health. In addition, zinc was also present in the drinking water. When the animals had completed five months of diet, Prof. Schraermeyer's lab including the rats moved to Tuebingen. The rats spent the last month of diet in Tuebingen and the local agent for animal protection was informed. Protocols compliant with § 4 paragraph 3 of the German law on animal protection were reviewed and approved by the “Einrichtung für Tierschutz, Tierärztlichen Dienst und Labortierkunde”. The experiments were performed in compliance with the ARVO statement for the use of animals in ophthalmic and visual research and all efforts were made to minimize the number of animals used and their suffering.

### 2) Animals

Adult (6–8 weeks old) Long Evans rats (LE, n = 16 eyes) were purchased from Harlan Winkelmann (Borchen, Germany). The animals were provided with tap water (zinc concentration less than 0.010 mg/l). The control animals (8 LE eyes) were fed *ad libitum* with normal nutrition containing 30 mg/kg zinc (control diet C1000, Altromin, Lage, Germany) and the zinc-deficiency (ZD) rats (8 ZD-LE eyes) were fed *ad libitum* with zinc deficient diet formulated to contain 5,9 mg zinc/kg diet (zinc deficient diet C1040, Altromin, Lage, Germany) for six months. ZD-LE rats were kept separately from LE rats so that “zinc contamination” in ZD-LE rats due to coprophagia can be excluded. The rats were handled and monitored either by the investigators or by the husbandry staff several times each week and no noticeable signs of illness such as loss of appetite, weight loss, diarrhoea, nasal or ocular discharge, skin or hair abnormalities, lethargy etc. were observed during or after the low zinc diet period. For light, fluorescence and electron microscopy, three LE control eyes and four ZD-LE eyes were used. For immunohistochemistry, five LE eyes and four ZD-LE eyes were investigated.

### 3) Preparation and embedding for light and electron microscopy

The rats were euthanized, they were first deeply anaesthetized by intraperitoneal injection of 100 mg/kg of ketamine (WDT, Garbsen, Germany) and 80 mg/kg of sedaxylan (WDT, Garbsen, Germany) following by transcardial injection of T61 (Intervet, Unterscleißheim, Germany). After enucleation, the eyes were cleaned of the orbital tissue and, after removal of the cornea, they were fixed overnight at 4°C in 2% glutaraldehyde in 0.1 M cacodylate buffer (pH 7.4) containing 100 mM sucrose. After washing with cacodylate buffer, areas of interest in flat mount preparations were excised and post-fixed with 1% osmium tetroxide in 0.1 M cacodylate buffer at room temperature for 1 h. Dehydration was then started by a series of incubations in 30%, 50%, and 70% ethanol. The samples were stained with saturated uranyl acetate. Dehydration was continued by incubations in 70%, 80%, 95% ethanol, absolute ethanol, and propylene oxide. The samples were then embedded in Epon (SPI-Pon™812 Epoxy Embedding Kit, SPI supplies, West Chester, PA). For EDX measurements, staining with osmium and uranylacetate was omitted.

### 4) Light microscopy and quantification of large pigmented cells

Semithin epon sections were stained with toluidine blue and examined under a light microscope (Axioplan2 imaging®, Zeiss, Göttingen, Germany). Images were obtained using a CCD camera connected to a personal computer by using an objective with a 40× magnification. The number of large pigmented cells observed in the choroid of ZD-LE rats was counted. Cells with a diameter larger than 3 µm were measured in an area spanning from the optic nerve to the ciliary body.

### 5) Electron microscopy and lipofuscin/melanosome quantification

Ultrathin epon sections (70 nm) from three LE control eyes and four ZD-LE eyes were post-stained with lead citrate and investigated under a transmission electron microscope (TEM, model 902 A, Carl Zeiss). For statistical analysis, the areas occupied by lipofuscin granules were quantified in 40 micrographs from four ZD-LE eyes and 30 micrographs from three LE eyes. These pictures were printed at final magnification of 20000 fold. As lipofuscin is not identical in all tissues, the functional definition we used in this study of the RPE was as follows: lipofuscin is a type of intracellular granule which appears in the electron microscope as a membrane bound body with heterogeneous staining generally darker than the cytosol and not identifiable as another cellular organelle e.g. not as dark as melanosomes. In particular, we excluded melanolipofuscin and quantified the classically appearing homogeneous lipofuscin in the general category “lipofuscin”. Melanin granules are easily and reliably distinguished from lipofuscin in the electron microscope, in that melanosomes are uniformly electron dense (black). Nothing with this staining property was included in the category of lipofuscin. A melanolipofuscin granule is one with a black core, surrounded by what appears to be normal lipofuscin. Lipofuscin granules and melanosomes can be easily identified by combining EDX and EELS (electron-energy-loss-spectroscopy) analysis in the TEM [54]. Melanosomes contain a significant amount of nitrogen (N) and no measurable phosphor (P) mole fractions while lipofuscin contains significant P mole fractions but N is at or below the minimum detectable mole fractions (MDMF) [54]. Accordingly, we analyzed typical melanosomes, melanolipofuscin and lipofuscin granules of a ZD-LE and a LE control rat by combined EDX and EELS analysis and used equivalent granules in the quantification experiments (see also Biesemeier et al. in prep). For lipofuscin quantification, image analysis software (iTEM, Olympus Soft imaging Solutions, Münster, Germany) was used. For each image, the total area of RPE cytoplasm was determined. Nuclei were not included in this measurement since it was assumed that they did not contain lipofuscin. Apical microvilli and extracellular space in the region of the basal infoldings were also excluded. The included area totalled from about 19–25 µm^2^ per image. Each lipofuscin granule was traced and its area determined in all images. The area fraction of lipofuscin is expressed as area in µm^2^ occupied by lipofuscin per 1000 µm^2^ RPE cytoplasm.

For the quantification of melanosomes in the RPE cells and of the area of the microvilli, 10 to 12 images with 7000× magnification which covered an area of about 170 µm in length (measured at Bruch's membrane) were used in all four ZD-LE rats and all three LE rats.

### 6) EDX spectroscopy in the TEM

Sections, approximately 120 nm thick, were mounted on aluminium grids. EDX spectra of melanosomes in non-stained sections were acquired on a Zeiss 912 Omega Analytical transmission Electron Microscope (AEM) equipped with an Omega energy filter, a 2 k*2 k CCD camera and an Oxford EDX detector at a magnification of 12500. Sample preparation and EDX spectrum acquisition were performed as described in detail elsewhere [54]–[56]. Spectra were quantitatively analysed by the INCA software (INCA, 2001), using the standardless Cliff-Lorimer k-factor method. The k- factors were those used in [56], the net Zn-Kα peak intensity was used for quantification. For high accuracy quantification, we only used spectra with minimum net counts for O being >15000. Therefore, the MDMF for Zn was less than 0.02 at%. The calculations of the MDMF were performed as described in [55]. A more detailed analysis of the investigated tissues by energy-filtered AEM will be presented elsewhere (Biesemeier et al., unpublished data).

### 7) Fluorescence microscopy

The eyes were fixed in formalin. A circular slit was cut at the limbus in order to immerse the inner eye with the fixation fluid. The eyes were then embedded in paraffin wax, cut to 5 µm sections, and de-paraffinized according to standard procedures. Fluorescent micrographs from paraffin sections were obtained from areas along the RPE layer. Auto-fluorescent lipofuscin and melanolipofuscin granules were photographed using a fluorescence microscope (Zeiss Axioplan2 imaging, excitation 370/36 nm, emission 575/15 nm) and an objective with a 60× magnification connected to a personal computer equipped with a CDD camera. Since the granules could not clearly be separated from each other and lipofuscin cannot be differentiated from melanolipofuscin, the quantification of lipofuscin granules was only performed using electron microscopy.

### 8) Immunohistochemistry

Paraffin sections were pre-treated with Pronase (Sigma) and were then incubated with a primary monoclonal mouse anti-rat ED1 antibody (Serotec, MCA341B, 1∶100) diluted with DAKO Diluent for blocking over night, followed by administration of the secondary Alexa 488 goat anti-mouse antibody (Molecular Probes, 1∶500), and mounted with DAKO Fluorescent Mounting Medium. The sections were examined using a fluorescence microscope (Zeiss Axioplan2 imaging, excitation 450–490 nm, emission 515–565 nm) by using an objective with a 60× magnification. All photographs were taken with the same camera settings (brightness, contrast, etc) to allow a comparison of staining intensity.

### 9) Statistical analysis

Statistical significance was determined by using the Mann-Whitney test for the ultrastructural evaluation of lipofuscin granules and the Student's t test for the quantification of large pigmented cells in the choroid of the animals. Values are reported as mean ± SEM. P≤0.05 was considered statistically significant.
